# Clinical Feature and Genetics in Rett Syndrome: A Report on Iranian Patients

**Published:** 2019

**Authors:** Parvaneh KARIMZADEH, Majid KHEIROLLAHI, Seyed Massoud HOUSHMAND, Sepideh DADGAR, Omid ARYANI, Omid YAGHINI

**Affiliations:** 1 **Pediatric Neurology ResearchCenter, Research Institute forChildren’s Health, Shahid Beheshti University of MedicalSciences, Tehran, Iran**; 2 **Pediatric NeurologyDepartment, Mofid Children’s Hospital, Faculty of Medicine, Shahid Beheshti University of Medical Sciences, Tehran, Iran**; 3 **Department of Medical Genetics, Pediatric Inherited Diseases Research Center, Research Institute for Primordial Prevention of Non-communicable Disease, Isfahan, Iran.**; 4 ** Department of Genetics and Molecular Biology, School of Medicine, Isfahan University of Medical Sciences, Isfahan, Iran.**; 5 **Department of Medical Genetic Medical Center and Faculty, Member of NIGEB, Tehran, Iran**; 6 **Pediatric Neurology, Child growth and Development research center, Research Institute for Primordial Prevention of Noncommunicable Disease of Medical Genetic, Isfahan University of Medical Sciences. Isfahan, Iran.**

**Keywords:** Rett Syndrome, MECP2, Genetics, Iran

## Abstract

**Objectives:**

Rett syndrome is characterized by normal development for the first 6-18 months of life followed by the loss of fine and gross motor skills and the ability to engage in social interaction. In most patients, mutations are found in methyl CpG-binding protein 2 (MECP2) gene. We investigated the relation between Rett clinical diagnosis and mutations in MECP2.

**Materials & Methods:**

Children suspected of Rett syndrome were invited to participate in this study. Twenty-three patients from the Mofid Hospital, Tehran, Iran suffered from classic Rett syndrome diagnostic criteria were enrolled in 2012. The severity of symptoms was assessed for all of them. The peripheral blood samples were collected in EDTA tubes and the genomic DNA was extracted using standard salting out method. The mutation of MEPC2 gene was studied using DNA sequencing method.

**Results:**

Overall, 11(47.8%) patients had MECP2 gene mutation, while 12 cases (52.2%) had no mutations. Changes in genetics were associated with phenotypical manifestations. The most prevalent mutation was p.v288 mainly associated with partially or uncontrolled seizures.

**Conclusion:**

For the first time, we studies the Rett syndrome in terms of clinical manifestations and genetic changes in Iran.

## Introduction

Rett syndrome is a X-linked progressive brain developmental disorder and one of the most common causes of mental retardation in females first described in the 1960s by Andreas Rett (1). Girls affected with classic form of Rett syndrome (RTT) seem to have normal development for 6 to 18 months. This course then followed by the loss of acquired fine and gross motor skills and the ability to engage in social interaction. Abnormal stereotypic hand movements may also occur. Rett syndrome primarily affects females and has an incidence of 1:10000 at birth until the age of 12 years (2).

Rett syndrome has two main types: classic and atypical. These two types may be characterized by their symptoms or specific gene mutations, and most patients with Rett syndrome have a classic form (3).

Mutations in the X-linked gene methyl CpG-binding protein 2 (MECP2) have been found in the majority of patients. MeCP2 is a protein that is critical for normal brain function. This protein is involved in maintaining synapses between nerve cells (4).

The mutations in the gene encoding *MECP2 *are associated with rare familial cases of RTT as well as in the usual sporadic cases of typical RTT (4). Using modern mutation detection tests, n 70%-80% of patients with typical RTT mutations are found in *MECP2 *(5). In addition to RTT, mutations in *MECP2 *have also been identified in cases with no clinical features of RTT. 

Mutations are in the X-linked gene MECP2, which encodes MeCP2 protein. MeCP2 is a member of family of methyl-CpG-binding domain proteins (MBD), but with their unique differences that help put it apart from the group. More than 600 MECP2 mutations have been identified, including missense, nonsense, frameshift and large deletion mutations that are pathogenic effect (RettBase; http://mecp2.chw.edu.au/). Most of these changes in MECP2 gene cause RTT in heterozygous females, but there is allelic heterogeneity in this disorder and a range of MECP2 mutations associated with variable phenotypic outcomes, including milder forms of learning disability and, rarely, autism, are also known (4, 6).

*MECP2* has two functional domains, a methyl-cytosine-binding domain (MBD) with composition of 85 amino acids and a transcriptional repression domain (TRD) which has 103 amino acids. The MBD domain binds to the methylation sites of CpG in the DNA strands and then TRD region makes reaction with SIN3A to utilize histone deacetylases (HDAC). There are also two high mobility group protein-like domains. The unusual, repetitive sequences are found at the carboxyl terminus (7, 8).

At one end of the spectrum, asymptomatic female carriers are found in familial RTT (11). Skewing of X chromosome inactivation (XCI) in these individuals allows them to have a normal presentation. At the other end of spectrum, boys with *MECP2 *mutations are confronted with severe early postnatal encephalopathy, early death, and absence of the distinctive clinical features of RTT (9).

In addition to *MECP2* mutations, cyclin-dependent kinase-like 5 (CDKL5) and Netrin G1, two other genes have recently been known in patients with clinical phenotype of Rett syndrome (10-14).

At present, a variety of clinical trials are done in RTT. As suggested in previous comprehensive studies conducted by Rett Search Consortium, clinical trials and other research studies to use a set of guidelines for classifying the disease (15). First, all individuals should be carefully reviewed and categorized clinically based on revised clinical criteria. The clinical diagnosis for all participants should be clearly stated in each publication. Second, comprehensive and complete genetic testing for mutations in *MECP2 *should be performed for all participants. This would include the sequencing of the coding region (15). 

We felt that Rett syndrome patients were not well studied in Iran. Therefore, we examined these patients based on the two main principals mentioned above to determine the spectrum of mutation in patients with Rett syndrome.

## Materials and Methods

**Patients and samples**

Twenty-three female children suspected of Rett syndrome referred to Neurology Outpatient Clinic, Mofid Hospital, Tehran, Iran in 2012 were invited to take part in this study. Referred individuals should have had a history of a period of relatively normal development after birth, followed by regression of developmental skills including the use of volitional hand, as well as reduced the speed of head circumference growth. The patients were enrolled in this study after observing the diagnostic criteria of classic Rett syndrome. The diagnosis of RTT was based exclusively on a set of clinical criteria derived from expert consensus (Table 1) (15). 

All examinations were performed by an expert pediatric neurologist. If any of them did not cooperate in the ongoing research, they were excluded from the study. For all included patients, severity of symptoms was assessed by a questionnaire. Locomotion, onset of signs and symptoms, seizure, head circumference growth, thrive, hand use, communication abilities, autonomic system disorders, EEG, scoliosis and self-abuse were graded from 0 to 3 to reflect severity of the signs and symptoms (Table 2). 

Informed consent was obtained from all the parents of patients for participation in this study.

***Molecular Methods***

*MEPC2* gene mutation was studied by DNA sequencing method. We collected blood sample from suspected patients referred to genetic laboratory. The genomic DNA was extracted from peripheral blood in EDTA tubes by standard salting out method. DNA purity was assessed with a spectrophotometer and calculated by ratio of the DNA optical density (OD 260) and protein optical density (OD 280). DNA yield was calculated from DNA optical density (OD 260) for clean DNA samples. To avoid errors derived from *Taq* PCR polymerase, PCR was performed using PFU DNA polymerase (Fermentas, St. Leon-Rot, Germany). Finally, the reaction products were sequenced and examined by Chromas program.

***Statistics***

Having gathered patients’ data and molecular results, descriptive correlation between genotype and phenotype was finally investigated.

## Results

Of 27 patients who met the inclusion criteria, 23 accepted to participate in the study. The mean age of 23 included patients in study was 5.2 yr with SD: ±2.13. The minimum and maximum age was 2, and 10 yr, respectively. The age of onset for signs and symptoms was ranged mostly less than 18 months (87%) and only 3 of 23 patients (13%) had shown their signs and symptoms in the range of 18-30 months. According to the chart brought in methods section, the severity of signs and symptoms were defined from 0 (less sever) to 3 (most sever) pluses (Table 3).

In addition, frequencies for each of signs or symptoms were calculated. Of 23 included patients, 3 could walk without help, 12 needed help, 4 needed devices to walk and 4 were disabled. Five patients were affected by uncontrolled Seizure, 4 had partially controlled and 9 had controlled seizure, meanwhile five Rett syndrome patients had not any complaint of seizure. Head circumference, thrive, hand use, communication abilities, autonomic system disorders, EEG, Scoliosis and self-abuse descriptive results are brought in Table 4. Regarding *MECP2* gene mutation, 11 patients (47.8% of total included patients) had this mutation meanwhile 12 (52.2%) had no mutation.

Of all 13 patients who showed epilepsy, *MECP2* positive patients were more prone to have epilepsy. Eight epileptic patients had *MECP2* mutation and the remaining 5 had not any evidence for *MECP2* mutation. However, severity of epilepsy (uncontrolled seizures) was not related to *MECP2*. Similar results were seen in EEG abnormalities.

Hand use, psychomotor retardation and communication problems were seen in all patients either in *MECP2* positives or negatives. Autonomic dysfunction was seen in 7 patients that only one of them had *MECP2* mutation and other 6 ones had no evidence of mutation.

**Figure 1:**
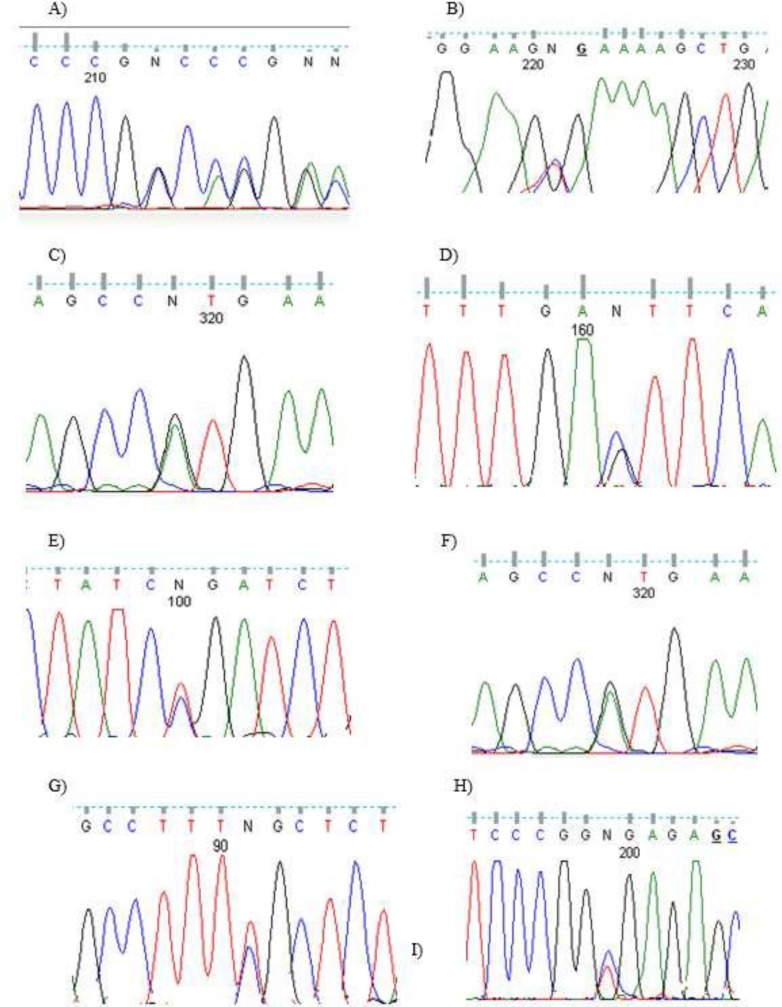
Electrophoretogram of patients. Refer to the text and Table 5 for an explanation of the details of each mutation

*MEPC2* gene mutation was studied for each patient too. Ten patients showed evidence of nucleotide changes including missense, nonsense or frameshift. The rest of patients did not show any evidence of changes. The detail of the changes detected for each patient is brought in Table 5 and Figure 1.

Change in genetics is in association with phonotypical manifestations. The most prevalent mutation was p.v288 which is mostly in association with partially or uncontrolled seizures. Moreover, the patients affected with this mutation show severe EEG abnormalities. In addition, we found new frameshift mutation in this study.

**Table 1:** Rett syndrome diagnostic criteria

Requirements Consider diagnosis when postnatal deceleration of head growth obseved		
Required for typical or classic RTT	Required for atypical or variant RTT	
1.A period of regression followed by recovery or stabilization 2.All main criteria and all exclusion criteria 3.supportive criteria are not required.although often present in typical RTT	1.A period of regression followed by recovery or stabilization 2.at least 2 of the 4 main criteria 3.5out of 11 supportive criteria	
Criteria		
Main criteria	1. Partial or complete loss of aquired purposeful hand skills 2. Partial or complete loss of aquired spoken language 3. Gait abnormalities:impaired or absence of ability 4. Stereotypic hand movements such as hand writing/squeezing, clapping/tapping,mouthing and washing/rubbing automatism	
Exclusion criteria for typical RTT	1.Brain injury secondary to trauma(peri- or postnataly),neurometabolic disease,or sever infection that causes neurological problems 2.Grossly abnormal psychomotor development in first 6 months of life	
Supportive criteria for atypical RTT	1. Breathing disturbance when awake 2. Bruxism when awake 3. Impaired sleep pattern 4. Abnormal muscle tone 5. Peripheral vasomotor distrurbance 6. scoliosois/kyphosis	7. Growth retardation 8. Small cold hands and feet 9. Inappropriate laughing/screaming spells 10. Diminshed response to pain 11. Intense eye communication”eye pointing”

**Table 2:** Signs and Symptoms severity assessment questionnaire

	0	1 plus	2 pluses	3 pluses
Signs and Symptoms age of onset	No	After 30 months	18-30 months	Less than 18 months
Locomotion	Without help	With help	With device	Disable to walk
Seizure	No	Under control	Partially controlled	Uncontrolled
Head circumference growth	Normal	Less than 2SD	Less than 3SD	Less than 4SD
Thrive	Normal	Mild FTT	Moderate FTT	Sever FTT
Hand use	Proper	Grasping	Moving toward	Disable
Communication abilities	Proper			-
Autonomic system disorders	Normal	Cold hands	Cold hands and feet	-
EEG	Normal	Mild abnormal	Moderate abnormal	Severe abnormal
Scoliosis	No	Mild	Moderate	Severe
Self abuse	No	Occasional	Usual	-

**Table 3:** Signs and symptoms for each patient

**Patient number**	**Thrive**	**Psychomotor regression**	**Head growth**	**Locomotion**	**communication**	**Autonomic dysfunction**	**Hand use**	**Self abuse**	**seizure**	**EEG**	**Scoliosis**	**Genetic mutation**
1	++	+	+	-	+	-	+	+	-	-	+	N^*^
2	-	+	+	+	++	-	++	-	++	++	-	Y^**^
3	+	+	++	+	++	+	++	+	-	-	+	N
4	-	+	-	+	+	-	+	-	+	+	-	Y
5	++	+	+	++	+	+	+	++	+++	+++	-	N
6	-	+	+	-	+	-	+	+	++	++	-	N
7	-	+	+	+	+	++	+	-	+	+++	-	N
8	+	+	+	+	+	+	++	-	+	++	-	Y
9	-	+	+	+	+	-	+	-	+	-	-	N
10	-	+	+	++	+	+	++	+	-	+	-	N
11	-	+	+	+++	+	-	+	-	-	-	-	N
12	-	+	+	+	+	-	+	-	-	-	-	Y
13	++	+	-	++	++	++	+++	+	+	+++	+++	N
14	++	+	++	+++	++	-	++	++	+++	+++	-	Y
15	-	+	++	+	+	+	+	-	+++	++	+	N
16	-	+	+++	+++	++	-	++	+	-	-	-	Y
17	++	+	+	++	+	-	++	+	+++	-	-	N
18	-	+	+	+	+	-	+	-	++	++	-	Y
19	-	+	-	+	++	-	+	+	+	+	-	N
20	-	+	+	-	+	-	++	+	+	+	+	Y
21	++	+	+	+	+	-	++	+	++	+++	+	Y
22	-	+	++	+	++	-	+	-	+++	++	-	Y
23	-	+	+	+++	+	-	+	-	-	-	-	Y

* N stands for patients without genetic mutation ** Y stands for patients with genetic mutation

**Table 4:** Frequency of severity of signs and symptoms

	Description	Number (percent)	Description	Number(percent)	Description	Number (percent)	Description	Number (percent)
Locomotion (walking)	Without help	3(13%)	With help	12(52.2%)	With device	4(17.4%)	Disable to walk	4(17.4%)
Seizure	No	5(21.7%)	Under control	9(39.1%)	Partially controlled	4(17.4%)	uncontrolled	5(21.7%)
Head circumference growth	Normal	3(13%)	Less than 2SD	15(65.2%)	Less than 3SD	4(17.4%)	Less than 4SD	1(4.3%)
Thrive	Normal	15(65.2%)	Mild FTT	2(8.7%)	Moderate FTT	6(26.1%)	Sever FTT	0
Hand use	Proper	0	Grasping	13(56.5%)	Moving toward	9(39.1%)	Disable	1(4.3%)
Communication abilities	Proper	0		16(69.6%)		7(30.4%)	-	0
Autonomic system disorders	Normal	16(69.6%)	Cold hands	5(21.7%)	Cold hands and feet	2(8.7%)	-	0
EEG	Normal	8(34.8%)	Mild abnormal	4(17.4%)	Moderate abnormal	6(26.1%)	Severe abnormal	5(21.7%)
Scoliosis	No	17(73.9%)	Mild	5(21.7%)	Moderate	0	Severe	1(4.3%)
Self abuse	No	11(47.8%)	Occasional	10(43.5%)	Usual	2(8.7%)	-	0

**Table 5:** Gene mutation was studied for each patient

Patient’s number	Nucleotide change	Amino acid change	Type of seq. change	References	Figure
18	c.750_750delCinsTCAGGAAGCTT	p.P251fs	Frame shift combined insertion and deletion	(24)	1A
21	c.763C>T	p.R255X	Nonsense	(25-33)	1B
2,14,22	c.862G>A	p.V288M	Missense	(34)	1C
23	c.468C>G	p.D156E	Missense	(35-41)	1D
20	c.880C>T	p.R294X	Nonsense	(18, 25-31, 42)	1E
8	c.473C>T	p.T158M	Missense	(18, 25-30, 42)	1F
4	c.397C>T	p.R133C	Missense	(4, 17, 25, 26, 29-31, 36, 37, 42-55)	1G
12	c.502C>T	p.R168X	Nonsense	(17, 18, 24-32, 37, 38, 42, 43, 45-49, 53-57)	1H

## Discussion

In this study, we reported the results of mutational analysis for *MECP2* gene in Iranian affected RTT girls. To our knowledge, this was the first time that Rett patients were studied in both clinical manifestations and genetic changes in Iran. About 47% of patients participated in our study showed *MECP2* mutation including missense, nonsense or frame shift.

The rate of mutation detection in our study is equal to some previously reported rates such as the 50% detection rate. It confirms the major role of *MECP2* gene mutation in etiology of Iranian patients too (16). On the other hand, there is a difference comparing to findings of another study where nearly 70%-80% of females with Rett’s syndrome had these mutations within the *MECP2* gene (5).

Of note, *MECP2* positivity differs in various countries, as De novo mutations were found in 60% of the patients in Spain which is closer to our findings (17). This difference is also referred to the limited number of patients studied in such studies and a series of patient who refused to participate like happened in our research. This reason could explain when comparing the results of a study in Japan in which 19 disease mutations (73%) were identified in 26 Japanese Rett patients (18).

The mutational diversity in our patients is consistent with the findings of other studies (16-18). In our study, about 10% of mutations were intragenic deletions or complex rearrangements that lead to frameshifts. In our study, of 10 *MECP2* positive mutations, we detected one case with frameshift in the C-terminal region. 

Moreover, the majority of mutations was C>T transitions which were nearly 70% of all identified mutations. Similarly, the majority of mutations in our study was C>T transitions too which was about 50% in another study (19).

The p.V288M amino acid change is identified as the most common mutational hotspot in our study. However, this mutation is not included in common formerly recognized hot spots. This is a new finding in patients with Rett syndrome in Iranian population.

In a previous study, 70% of the mutations within MECP2 were in eight hotspots which affect translation of the amino acids including R106, R133, T158, R168, R255, R270, R294, and R306 (20).

According to patients’ signs and symptoms, despite in some items like epilepsy and EEG abnormalities, *MECP2* positives showed slightly higher rates compared to those without mutations, and autonomic dysfunctions were more seen in *MECP2* negatives, but none of them revealed significant correlation between signs and mutations. Moreover, severity of signs and symptoms is not in relation with gene mutation and loci of mutation in our research. The only exception refers to sever EEG abnormality seen in patients with p.V288M amino acid change. 

Location and frequency of *MeCP2* ‘‘hotspot’’ mutations in RTT patients show that most of these mutations are point substitutes in nucleotides including R106W, arginine to tryptophan point mutation at residue 106; R133C, arginine to cysteine point mutation at residue 133; T158M, threonine to methionine point mutation at residue 158; R168X, arginine to stop codon at residue 168; R255X, arginine to stop codon at residue 255; R294X, arginine to stop codon at residue 294; R306C, arginine to cysteine point mutation at residue 306. The amino acids 207and 310 of TRD are involved in the repression of transcription of target genes, but the mechanism of TRD repression is unknown (21).

The study of genotype-phenotype correlation requires precise investigations in larger group of patients to determine correlation with various types of mutations.

Genotype-phenotype correlation has been investigated, but it is complex by *MECP2* gene X-chromosome inactivation. This inactivity allows a mother with a mutation of MECP2 to have a normal phenotype because of skewing of X-chromosome inactivation (22). In contrast to the problems with X-chromosome inactivation, many studies of genotype-phenotype correlations exist (17, 22, 23). More studies must be carried out to investigate more case to find better conclusions.

**In conclusion,** our results together with data reported by others allow general conclusions about the MECP2 mutational spectrum and growth/developmental manifestations or phenotypes.

## Author`s contribution

Parvaneh Karimzadeh: Substantial contributions to the conception or design of the work; or the acquisition, analysis, or interpretation of data for the work.

Majid Kheirollahi: Drafting the work or revising it critically for important intellectual content, final approval of the version to be published 

Seyed Massoud Houshmand: Final approval of the version to be published.

Sepideh Dadgar: Agreement to be accountable for all aspects of the work in ensuring that questions related to the accuracy or integrity of any part of the work are appropriately investigated and resolved

Omid Aryani: Agreement to be accountable for all aspects of the work in ensuring that questions related to the accuracy or integrity of any part of the work are appropriately investigated and resolved

Omid Yaghini: Drafting the work or revising it critically for important intellectual content, final approval of the version to be published (ORCID ID

All authors agreed to be accountable for all aspects of the work in ensuring that questions related to the accuracy or integrity of any part of the work are appropriately investigated and resolved.
